# After Traumatic Brain Injury, EPHA4 Enhances Endoplasmic Reticulum Stress to Promote M1 Microglial Polarization Through the MAPK Signaling Pathway

**DOI:** 10.1155/mi/5595023

**Published:** 2026-01-22

**Authors:** Yang Tan, Jing Xia, Mingwei Liu, Sangyang Deng, Haiying Wu, Chuanyun Qian

**Affiliations:** ^1^ Emergency Medicine, The First Affiliated Hospital of Kunming Medical University, Kunming, Yunnan, China, kmmc.cn; ^2^ Department of Emergency, Dali Bai Autonomous Prefecture People’s Hospital, Dali, Yunnan, China; ^3^ Kunming Medical University, Kunming, Yunnan, China, kmmc.cn

**Keywords:** endoplasmic reticulum stress, EPHA4, M1 polarization of microglia, MAPK signaling pathways, MI-5595023, traumatic brain injury

## Abstract

**Background:**

Traumatic brain injury (TBI) is an important cause of disability and death worldwide. The development of neuroinflammation after TBI is related to the brain parenchyma. M1‐type microglia play important roles in this process, but the specific mechanism through which regulating microglia M1 polarization is still not fully understood. This study aimed to investigate the role of ephrin receptor A4 (EPHA4) in the M1 polarization of microglia after TBI.

**Methods:**

A TBI rat model was established by the controlled cortical impact (CCI) method, and M1 polarization of GMI‐R1 cells was induced by lipopolysaccharide (LPS) treatment. Target genes associated with the progression of TBI were screened by transcriptome sequencing; the expression of key genes and proteins was detected by real‐time quantitative PCR (RT‒qPCR), Western blot, ELISA, and immunofluorescence, and the damage of the rat brain tissue and the blood–brain barrier (BBB) was evaluated by hematoxylin‒eosin (HE) staining and Evans blue staining.

**Results:**

This study revealed that EPHA4 expression is upregulated in the brain tissue of TBI rats and that treatment with its inhibitor, KYL peptide, can improve the progression of TBI. KYL peptide intervention downregulated the levels of the proinflammatory cytokines and upregulated the levels of the anti‐inflammatory cytokines and inhibited the M1 polarization of microglia. The levels of the endoplasmic reticulum stress (ERS)‐related proteins and Ca^2+^ were upregulated in TBI rats and downregulated after KYL peptide treatment but subsequently increased after 123C4 treatment. Our results showed that EPHA4 promoted the M1 polarization of microglia by enhancing ERS. Notably, mitogen‐activated protein kinase (MAPK) signaling was significantly enriched in TBI. In vitro studies revealed that LPS treatment promoted the activation of MAPK signaling in GMI‐R1 cells. A mechanistic study revealed that EPHA4 may activate ERS by activating the MAPK signaling pathway and promote M1 polarization of microglia after TBI.

**Conclusion:**

Our study revealed that the EPHA4/MAPK axis may be a key regulatory factor in controlling microglial M1 polarization during brain injury. Blocking this signaling axis may represent a potential therapeutic approach for improving TBI.

## 1. Introduction

Traumatic brain injury (TBI) is a disease that involves the interruption or impairment of brain function caused by external physical forces [1], often leading to the development of a series of pathological events, such as long‐term chronic sequelae and neurodegeneration [2]. Mechanistically, both primary injury and secondary injury are related to the neuropathological progression of TBI. Primary injury occurs after mechanical stress, whereas secondary injury is the cascade of pathological responses induced by primary injury, including ion homeostasis imbalance, oxidative stress, inflammation, and cell death [3]. Neuroinflammation is among the most important cellular events after TBI [4]. Neuroinflammation usually leads to the accumulation of harmful metabolites in the brain parenchyma, which aggravates cerebral edema and promotes peripheral immune cell infiltration and neuroapoptosis [5]. Microglia are the main resident endogenous immune cells of the central nervous system and play important roles in regulating the neuroinflammatory process in TBI [6]. After TBI, resident microglia are rapidly activated to the inflammatory phenotype (M1) and are mobilized to the damaged area to remove debris and induce inflammation [7]. However, excessive activation of microglia is harmful and often leads to the aggravation of blood–brain barrier (BBB) damage and the malignant progression of the disease [8]. Therefore, this study will continue to investigate the specific mechanisms that regulate the M1 polarization of microglia after TBI.

Previous studies have shown that endoplasmic reticulum stress (ERS) is critical for microglial activation, and treatment with the exogenous ERS inducer tunicamycin can mediate the neuroinflammatory response through regulation of the microglial polarization state [9]. The ER is an important intracellular organelle in which reactions such as protein synthesis, maturation, folding, modification, and denaturation occur [10]. ERS occurs when protein folding equilibrium is disrupted. Under ERS, the dissociation of glucose‐regulated protein 78 (GRP78) can restore cellular homeostasis by promoting the activation of protein kinase RNA‐like ER kinase (PERK), inositol requiring enzyme (IRE1), and activating transcription factor 6 (ATF6) [11]. However, uncontrolled ERS further activates C/EBP homologous protein (CHOP) to trigger neuronal apoptosis after TBI [12]. The inhibition of ERS has been confirmed to be an effective strategy for improving neuroinflammation and white matter injury induced by M1‐type microglia after TBI [13]. However, the molecular mechanisms underlying the influence of ERS on the M1 polarization of microglia are still not fully understood.

Ephrin receptor A4 (EPHA4) is a member of the EPHA receptor family and plays an important role in hippocampal neurogenesis [14]. Previous studies have revealed an association between EPHA4 and Alzheimer’s disease [15] and amyotrophic lateral sclerosis [16]. EPHA4 is also key for cerebrovascular function [17]. In TBI, EPHA4 leads to abnormal neurogenesis, memory impairment [18], cerebral cortical injury [19], and BBB injury [20]. Importantly, EPHA4 may also be related to the regulation of cellular ERS [21]. However, whether EPHA4 affects the M1 polarization of microglia after TBI through the regulation of ERS needs further study.

In addition, mitogen‐activated protein kinases (MAPKs) belong to the serine‐threonine kinase family and are involved in cell biological behaviors such as cell proliferation, differentiation, apoptosis, inflammation, and metabolism [22]. The activation of MAPK signaling is associated with the development of neurodegenerative diseases [23]. In TBI, the activation of MAPK signaling is also an important cause of the development of neuroinflammation‐related sequelae after TBI [24]. Similarly, MAPK signaling‐mediated chronic inflammation can also induce long‐term cognitive and mood disorders after TBI [25]. In addition, the MAPK signaling pathway affects the neuroinflammatory response by regulating microglial polarization [26]. Notably, the activation of the MAPK signaling pathway is regulated by EPHA4 [27]. Therefore, we speculated that EPHA4 may affect the M1 polarization of microglia after TBI through the MAPK signaling pathway.

In summary, the purpose of this study was to investigate the function and mechanism of EPHA4 in microglial M1 polarization after TBI and to identify potential intervention targets for improving the progression of TBI.

## 2. Materials and Methods

### 2.1. Animal Experiments

Male SD rats (6–8 weeks, 220–250 g) were purchased from Hunan Slack Jingda Experimental Animal Co., Ltd., (China). All experimental rats received 1 week of adaptive feeding before TBI modeling. A rat TBI model was induced by the controlled cortical impact (CCI) method [28]. Briefly, a right parietal craniotomy (2 mm lateral to the midline and 1 mm anteroposterior; 4 mm diameter) was performed after the rats were anesthetized with 5% isoflurane. After that, using a pneumatically driven CCI device (AMS 201, AmScience, USA), a flat tip with a diameter of 2 mm was tapped vertically on the exposed cerebral cortex (velocity of 3.5 m/s for 150 ms with a depth of 1.5 mm). After the impact is complete, the bone flap is repositioned and sealed. As a control, rats in the sham group received craniotomy only and did not receive rod drop impact. After 1 day, 3 days, 7 days, 14 days, and 28 days of TBI induction, the rats were anesthetized with 3% pentobarbital sodium (40 mg/kg; Sigma‒Aldrich; intraperitoneal injection) and euthanized by cervical dislocation. Then, the brain tissues were collected for subsequent studies. In addition, to explore the effect of EPHA4 on TBI rats, the EPHA4 inhibitor KYL peptide (HY‐P2264, MCE, USA) was continuously delivered into the rats through an Alzet implantable capsule osmotic pump (1007D; Beijing Unique Biotechnology Co., Ltd., China) 6 h after TBI modeling. The intervention dose was 10 mg/kg, and the dosing regimen of the KYL peptide was described previously [19]. The 123C4 (HY‐P0177; MCE, USA), an effective, selective, and competitive receptor tyrosine kinase EPHA4 activator, was injected intraperitoneally into rats 6 h after TBI induction to activate EPHA4. The injection dose of 123C4 was 30 mg/kg once a day for 3 days. In addition, KYL peptide‐treated TBI rats were euthanized after 3 days of treatment, after which the brain tissue was isolated for subsequent examination. All animal experiments were approved by the Animal Experiment Ethics Committee of Kunming Medical University (kmmu20211255) and adhered to the ARRIVE guidelines.

### 2.2. Transcriptome Sequencing

After 3 days of TBI induction, the rat brain tissue was isolated, and total RNA was extracted with TRI reagent (T9424; Sigma‒Aldrich, USA). RNA was subsequently reverse‐transcribed into cDNA using a first‐strand cDNA synthesis kit (11483188001; Sigma‒Aldrich, USA). Then, the cDNA was sent to Novogene (China), and transcriptome sequencing was performed.

### 2.3. Measurement of Brain Tissue Water Content

To determine the water content of the brain tissue, the brain tissue was removed from each rat, and the weight was recorded as the wet weight. The brain tissue was then placed in a 100°C oven for 24 h, after which the weight was recorded again as the dry weight. The water content of the brain tissue was calculated by the formula (wet weight – dry weight)/wet weight × 100%.

### 2.4. Evans Blue Staining

Rat BBB permeability was evaluated by Evans blue staining. A 4% Evans blue staining solution (ST3273; Beyotime, China) was injected into the tail vein of the rats at a concentration of 3 mg/kg. Three hours after injection, the rats were sacrificed, and the chest was opened. A 0.9% sodium chloride solution (containing heparin) was injected into the apex of the heart, and the right atrium was cut until the outflow of the right atrium was clear. Brain tissue was then isolated, and the leakage of Evans blue was observed.

### 2.5. Hematoxylin‒Eosin (HE) Staining

Rat brain tissue was fixed in 4% paraformaldehyde and embedded in paraffin, and sections with a thickness of 5 μm were cut from the wax blocks. After that, staining was performed using an HE staining kit (G1076; Servicebio, China) according to the manufacturer’s instructions. After the sections were dehydrated with ethanol and permeabilized in xylene, the lesions in the rat brain tissue were observed under a microscope.

### 2.6. Cell Experiments

Rat microglia GMI‐R1 cells (HTX3594; RRID: CVCL_W175) were purchased from Otwo Biotech, Inc. (Shenzhen, China). The cells were subjected to mycoplasma testing, free of contamination, and accurately identified by short tandem repeat (STR). GMI‐R1 cells were grown in DMEM (11965092; Gibco, USA) supplemented with 10% fetal calf serum (A5256701; Gibco, USA) and 1% penicillin‒streptomycin (P1400; Solarbio, China). To induce M1 polarization in GMI‐R1 cells, the cells were treated with 10 μg/mL lipopolysaccharide (LPS, HY‐D1056, MCE, USA) in media for 24 h. Furthermore, to explore the effect of the EPHA/MAPK signaling axis on the M1 polarization of GMI‐R1 cells, the EPHA4 inhibitor KYL peptide (20 μM; HY‐P2264; MCE, USA) and the MAPK activator C16‐PAF (1 μM; HY‐108635; a potent MAPK activator) were added to GMI‐R1 cells for 24 h to inhibit EPHA4 expression and activate MAPK signaling. In addition, 100 μM EPHA4 activator 123C4 (HY‐P0177; MCE, USA) and 2.5 mM ERS inhibitor 4‐phenylbutyric acid (4‐PBA; HY‐A0281; MCE, USA) were used to treat GMI‐R1 cells for 24 h to activate EPHA4 and inhibit ERS.

### 2.7. Real‐Time Quantitative PCR (RT‒qPCR)

The methods for RNA extraction and cDNA preparation were the same as those described above for transcriptome sequencing. SYBR Green reagent (D7260; Beyotime, China) was used for RT‐qPCR, and the results were calculated by the 2^−ΔΔCt^ method. The primer sequences are listed in Table 1.

**Table 1:** Primer Sequence Information.

Gene	Primer sequence (5^′^‐3^′^)
EPHA4	F: CTACGGAATCGTTATGTGGG
	R: GCTTTGATCACATCTTGATTGG
β‐actin	F: GGTCAGGTCATCACTATCGG
	R: GGATTCCATACCCAGGAAGG

### 2.8. Western Blot Analysis

Total protein from rat brain tissues and GMI‐R1 cells was extracted using RIPA buffer (89901; Thermo Fisher, USA) supplemented with 1% protease inhibitors and phosphatase inhibitors and was assayed with a BCA protein concentration assay kit (PC0020; Solarbio, China) to quantify the protein concentration. After that, the total protein was separated by SDS‒PAGE and transferred to a PVDF membrane (88518, Thermo Fisher, USA). The membrane was incubated with diluted primary antibody at 4°C overnight. The primary antibodies used in this study were against EPHA4 (1:1000, 21875‐1‐AP, Proteintech, China), iNOS (1:1000, 22226‐1‐AP, Proteintech, China), COX2 (1:1000, 12375‐1‐AP, Proteintech, China), CD86 (1:1000, 13395‐1‐AP, Proteintech, China), Arg1 (1:2000, 16001‐1‐AP, Proteintech, China), CD206 (1:1000, 18704‐1‐AP, Proteintech, China), PERK (1:2000, 68482‐1‐Ig, Proteintech, China), p‐PERK (1:1000, 29546‐1‐AP, Proteintech, China), IRE1α (1:1000, PA5‐20189, Invitrogen, USA), p‐IRE1α (1:1000, PA5‐85738, Invitrogen, USA), ATF6 (1:2000, 24169‐1‐AP, Proteintech, China), CHOP (1:1000, 15204‐1‐AP, Proteintech, China), GRP78 (1:2000, 11587‐1‐AP, Proteintech, China), and ERK1/2 (1:1000, #4695, CST, USA), p‐ERK1/2 (1:2000, #4370, CST, USA), p38 MAPK (1:1000, #8690, CST, USA), p‐p38 MAPK (1:1000, #4511, CST, USA), JNK (1:1000, #67096, CST, USA), p‐JNK (1:2000, #9255, CST, USA), β‐actin (1:2000, ab8226, Abcam, UK). Afterward, the membrane was incubated with the corresponding secondary antibody for 1 h at room temperature, and the protein bands were visualized using an enhanced chemiluminescence kit (WBULP, Millipore, USA). Semiquantitative analysis of protein bands was performed using ImageJ software.

### 2.9. Immunofluorescence

After the cell culture was complete, the medium was discarded, and the cells were fixed with 4% paraformaldehyde for 20 min. Then, the cells were incubated with primary antibodies against iNOS (1:200; 22226‐1‐AP; Proteintech, China) and Arg1 (1:200; 16001‐1‐AP; Proteintech, China) at 4°C overnight. Afterward, the corresponding fluorescent secondary antibody was incubated at room temperature for 2 h. After nuclear staining with DAPI (C1002; Beyotime, China) for 15 min, the cells were observed, and images were captured under a fluorescence microscope. The mean fluorescence intensity was quantified using ImageJ software. The results of the experimental group were normalized based on those of the control group.

To explore the effect of TBI on the expression of EPHA4 in microglia, rat brain tissue was harvested after 3 days of TBI induction, and paraffin sections were prepared. The sections were incubated with anti‐EPHA4 (1:100; 21875‐1‐AP; Proteintech, China) and anti‐Iba1 (1:200; ab178846; Abcam, UK) primary antibody at 4°C. In addition, to explore the function of EPHA4 in microglial activation in TBI rat brain tissue, primary antibodies against Iba1 (1:100; ab178846; Abcam, UK), iNOS (1:200; 22226‐1‐AP; Proteintech, China), or Arg1 (1:200; 16001‐1‐AP; Proteintech, China) were added at 4°C. After incubating the sections with the primary antibody overnight, the sections were treated with the corresponding fluorescent secondary antibodies for 2 h at room temperature. Then, the nuclei were stained with DAPI staining solution (C1002; Beyotime, China) for 15 min, and the sections were placed under a fluorescence microscope to observe pictures. The number of positive cells was counted using the Image J software. A total of five animals per group were analyzed.

### 2.10. ELISA Experiment

ELISA kits (Solarbio, China) were used to measure the levels of interleukin 1β (IL‐1β, SEKR‐0002), IL‐6 (SEKR‐0005), tumor necrosis factor α (TNF‐α, SEKR‐0009), IL‐4 (SEKR‐0004), IL‐10 (SEKR‐0006), and transforming growth factor β1 (TGF‐β1, SEKR‐0012) levels in rat brain tissues and GMI‐R1 cells. Briefly, after the rat brain tissue homogenate and GMI‐R1 cell culture supernatant were harvested, the supernatant was collected by centrifugation at 5000× *g* for 10 min at 4°C, and the absorbance value of the samples at 450 nm was determined using a microplate reader.

### 2.11. Detection of Ca^2+^ Levels

Ca^2+^ levels in rat brain tissue and GMI‐R1 cells were measured using a Fluo‐3 AM probe (Solarbio, Beijing, China). Briefly, rat brain tissue and GMI‐R1 cells were treated with 0.25% trypsin to prepare single‐cell suspensions, and the cell density was adjusted to 1 × 10^6^/mL and incubated with the Fluo‐3 AM probe at a final concentration of 2 μM at 37°C for 30 min. Then, the cells were loaded onto a flow cytometer for analysis. The mean fluorescence intensity was calculated to reflect the Ca^2+^ levels within the cells. Furthermore, forward scatter (FSC) reflects the size of the cells, while side scatter (SSC) reflects the complexity or granularity of the cells. The samples were gated on the two‐dimensional graphs of FSC and SSC to eliminate debris and dead cells while retaining the population of living cells.

### 2.12. Statistical Analysis

All experimental data are expressed as the mean ± standard deviation (mean ± SD). Statistical analysis was performed on the experimental data using GraphPad Prism 8.1 software (GraphPad Software Inc., San Diego, CA, USA). All the experimental animals were randomly grouped. All group allocation, data collection, and analysis were conducted using a blinded manner. Five animals per group were used for each pathological experiment, and other five animals per group were used for mRNA, protein, and Ca^2+^ level detection experiments. No statistical methods were used to predetermine the sample sizes; however, the sample sizes were similar to that of our previous studies and those reported in previous academic research. Shapiro–Wilk test was used to evaluate the normal distribution of the data, Levene test was used to analyze the homogeneity of variance, and all data meet the statistical assumptions for equality of the variance and for normal distribution. A *t* test was used to compare the data between two groups, one‐way analysis of variance (ANOVA) was used to compare the data between multiple groups, and Tukey’s post hoc test was used to perform multiple comparisons. A *p*‐value <0.05 was considered to indicate statistical significance.

## 3. Results

### 3.1. EPHA4 Is Highly Expressed in Rat Brain Tissue After TBI and Is Associated With the Activation of Microglia

First, to screen target genes associated with the progression of TBI, we performed transcriptome sequencing. After data analysis, we found that EPHA4 was highly expressed in the brain tissue of TBI rats (Figure 1A,B). Next, we validated the expression of EPHA4 in vivo. The results showed that the expression of EPHA4 mRNA and protein in rat brain tissue was significantly upregulated after TBI and that the level of EPHA4 was greatest after 3 days of TBI (Figure 1C,D). Subsequent studies used a 3‐day intervention cycle to induce TBI in rats. Notably, previous studies have confirmed that EPHA4 is associated with the M1 polarization of microglia in ischemic brain injury [29]. Subsequently, immunofluorescence was used to detect the expression of EPHA4 in microglial cells of the rat brain tissue after 3 days of TBI. The results showed that the coexpression level of EPHA4 and Iba1 was significantly increased (Figure 1E). These results indicate that EPH4A expression is upregulated after TBI and is related to microglial activation.

Figure 1Transcriptome sequencing analysis of differentially expressed genes in rat brain tissues after TBI. (A) Analysis of differential gene expression via heatmap. (B) Analysis of differential gene expression via volcano map. (C) EPHA4 mRNA expression in rat brain tissue was detected by RT‒qPCR. (D) EPHA4 protein expression in rat brain tissue was detected by Western blot. (E) EPHA4 and Iba1 expressions were detected via immunofluorescence staining; scale bar: 20 μm. Molecular experiment (C,D), *n* = 5 rats/group; pathological experiment (E), *n* = 5 rats/group. Compared with the sham group,  ^∗∗^ *p* < 0.01,  ^∗∗∗^ *p* < 0.001.

**Figure:**
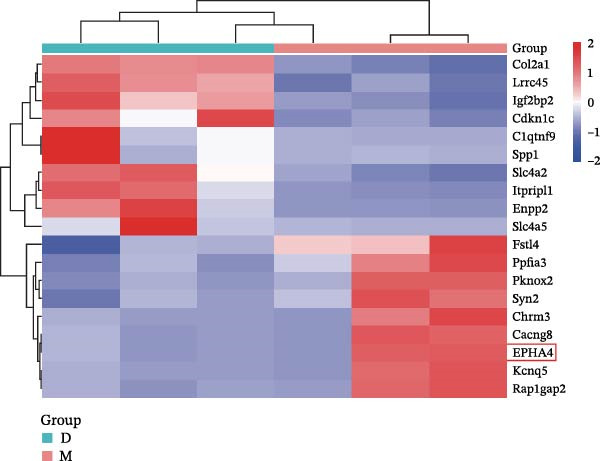
(A)

**Figure:**
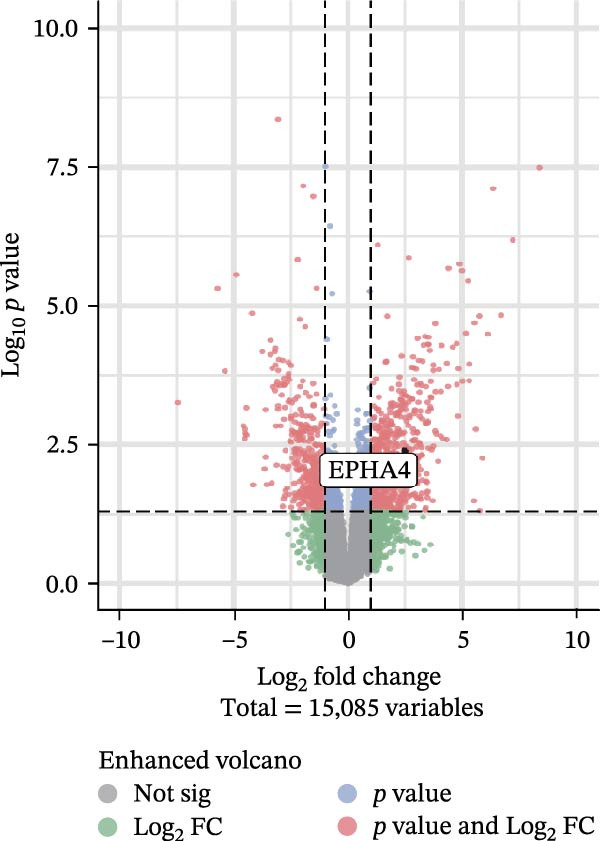
(B)

**Figure:**
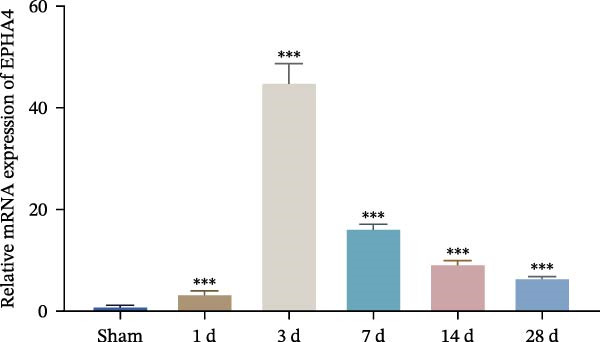
(C)

**Figure:**
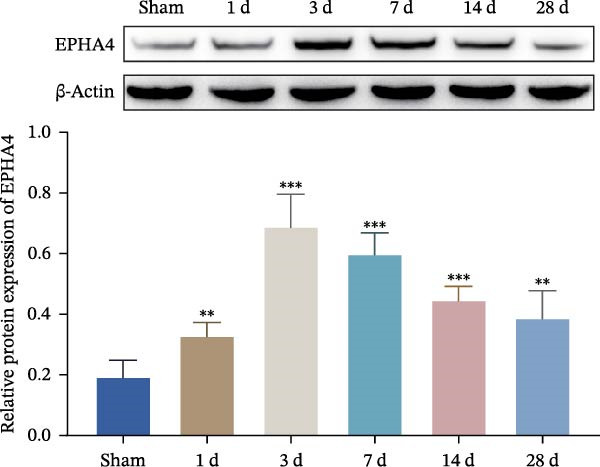
(D)

**Figure:**
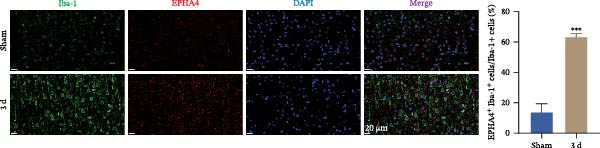
(E)

### 3.2. Inhibition of EPHA4 Inhibits the Progression of TBI in Rats

To explore the function of EPHA4 in the progression of TBI, we inhibited EPHA4 expression in TBI rats by supplementation with the EPHA4 inhibitor KYL peptide. We found that KYL peptide suppressed EPHA4 mRNA (Figure 2A) and protein (Figure 2B) expression in the cerebral cortex of TBI rats. Furthermore, TBI treatment increased the water content in the rat brain tissue, whereas after KYL peptide treatment, the water content in the rat brain tissue decreased somewhat (Figure 2C). The results of Evans blue staining revealed that TBI caused uncontrolled leakage in rat brain tissue, whereas the leakage improved after KYL peptide intervention (Figure 2D). HE staining of the lesions in the brain tissue revealed that the integrity of the brain tissue in the TBI‐treated rats was impaired but improved after KYL peptide treatment (Figure 2E). These results suggest that inhibition of EPHA4 expression can effectively attenuate the progression of TBI.

Figure 2Inhibition of EPHA4 expression attenuates BBB injury in rats after TBI. (A) RT‒qPCR detection of EPHA4 mRNA expression in rat brain tissue. (B) Western blot detection of EPHA4 protein expression in rat brain tissue. (C) Detection of water content in brain tissue. (D) Evans blue staining to assess the BBB permeability in rat brain tissue. (E) HE staining of rat brain tissues; scale bar: 500 μm. *n* = 5 rats/group. The same group of rats was used in the experiments A and B. Compared with the sham group,  ^∗∗^ *p* < 0.01,  ^∗∗∗^ *p* < 0.001; compared with the TBI group, ^###^ *p* < 0.001.

**Figure:**
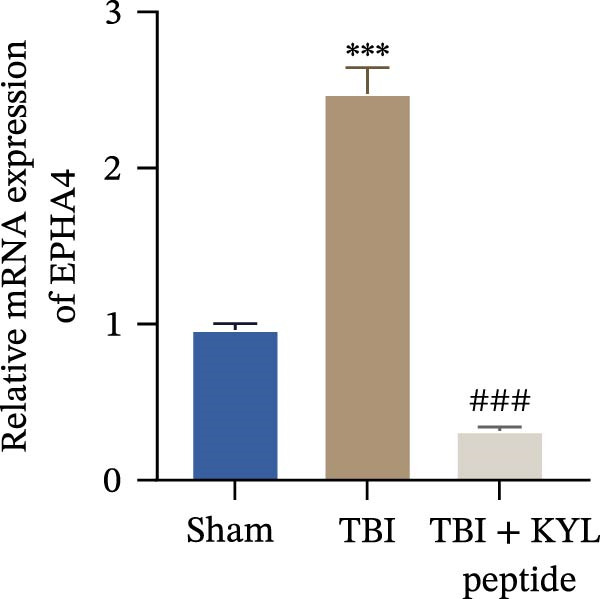
(A)

**Figure:**
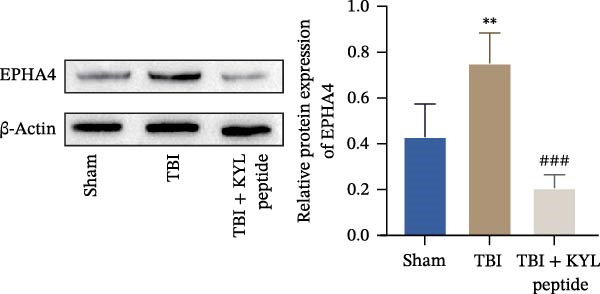
(B)

**Figure:**
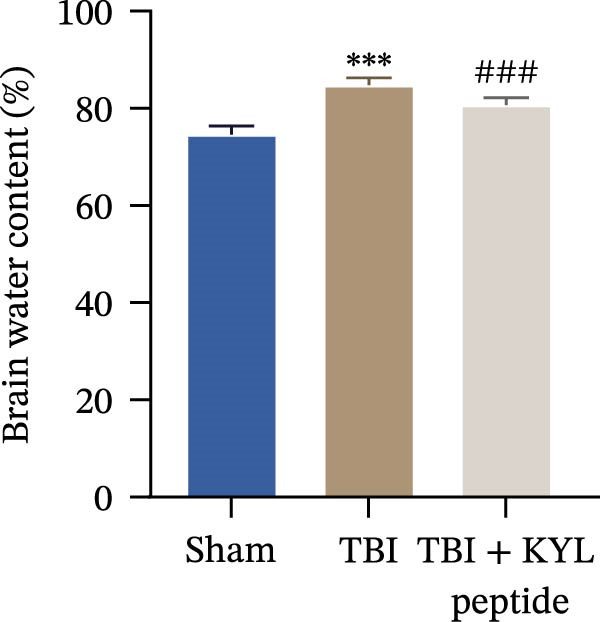
(C)

**Figure:**
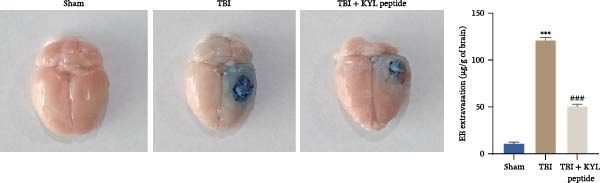
(D)

**Figure:**
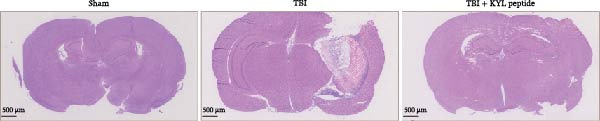
(E)

### 3.3. Inhibition of EPHA4 Inhibits M1 Polarization of Microglia in Rats After TBI

Next, we explored the role of EPHA4 in M1 microglial activation after TBI. ELISA revealed that after TBI, the levels of the proinflammatory cytokines IL‐1β, IL‐6, and TNF‐α increased, whereas the levels of the anti‐inflammatory cytokines IL‐4, IL‐10, and TGF‐β1 decreased in rat brain tissue. The abovementioned effects of TBI could be weakened by the addition of the KYL peptide (Figure 3A). Next, the levels of the M1 polarization markers iNOS, COX2, and CD86 and the M2 polarization markers Arg1 and CD206 in the rat brain tissues were detected by Western blotting. The results showed that TBI promoted the expression of iNOS, COX2, and CD86, while inhibiting the expression of Arg1 and CD206. After KYL peptide treatment, the expression levels of iNOS, COX2, and CD86 were downregulated, and the expression levels of Arg1 and CD206 were upregulated (Figure 3B). In addition, immunofluorescence staining revealed that the coexpression levels of Iba1 and iNOS were significantly decreased and that the coexpression levels of Iba1 and Arg1 were significantly increased in the brain tissues of TBI rats after KYL peptide treatment (Figure 3C). These results suggest that inhibition of EPHA4 expression can suppress the M1 polarization of microglia after TBI.

Figure 3Inhibition of EPHA4 expression suppresses the M1 polarization of microglia after TBI in rats. (A) ELISA detection of IL‐1β, IL‐6, TNF‐α, IL‐4, IL‐10, and TGF‐β1 levels in rat brain tissue. (B) Western blot detection of the M1 polarization markers iNOS, COX2, and CD86 as well as the M2 polarization markers Arg1 and CD206 in rat brain tissue. (C) Immunofluorescence detection of Iba1, iNOS, and Arg1 expression in rat brain tissues; scale bar: 20 μm. Molecular experiment (A, B), *n* = 5 rats/group; pathological experiment (C), *n* = 5 rats/group. Compared with the sham group,  ^∗∗∗^ *p* < 0.001; compared with the TBI group, ^#^ *p* < 0.05, ^##^ *p* < 0.01, ^###^ *p* < 0.001.

**Figure:**
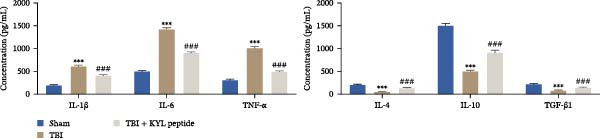
(A)

**Figure:**
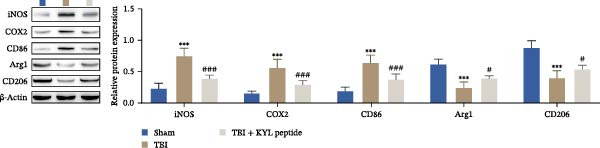
(B)

**Figure:**
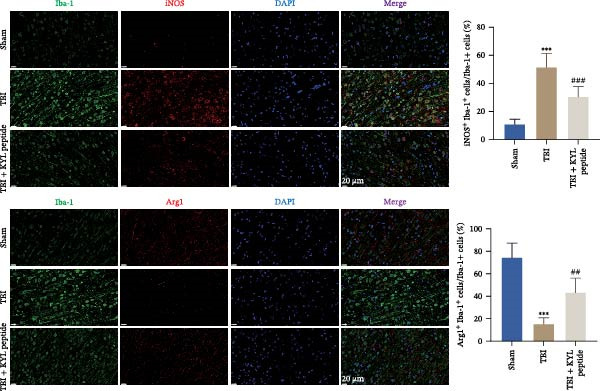
(C)

### 3.4. Inhibition of EPHA4 Suppresses LPS‐Induced M1 Polarization of Microglia

Subsequently, we explored the function of EPHA4 in the LPS‐induced M1 polarization of GMI‐R1 cells at the cellular level. Compared with the control group, LPS treatment upregulated the levels of IL‐1β, IL‐6, and TNF‐α in the supernatant of GMI‐R1 cells and downregulated the levels of IL‐4, IL‐10, and TGF‐β1. Further treatment with the KYL peptide attenuated the effect of LPS (Figure 4A). Furthermore, after KYL peptide intervention, the levels of iNOS, COX2, and CD86 were downregulated, and the levels of Arg1 and CD206 were upregulated in GMI‐R1 cells (Figure 4B,C). These results indicated that the inhibition of EPHA4 could inhibit the LPS‐induced M1 polarization of GMI‐RI cells.

Figure 4Inhibition of EPHA4 expression suppresses LPS‐induced M1 polarization of microglia. (A) ELISA detection of IL‐1β, IL‐6, TNF‐α, IL‐4, IL‐10, and TGF‐β1 levels in GMI‐R1 cells. (B) Western blot detection of M1 polarization markers iNOS, COX2, and CD86 as well as the M2 polarization markers Arg1 and CD206 in GMI‐R1 cells. (C) Immunofluorescence detection of the expression of iNOS and Arg1 in GMI‐R1 cells; scale bar: 10 μm. Compared with the control group,  ^∗∗∗^ *p* < 0.001; compared with the LPS group, ^#^ *p* < 0.05, ^##^ *p* < 0.01, ^###^ *p* < 0.001.

**Figure:**
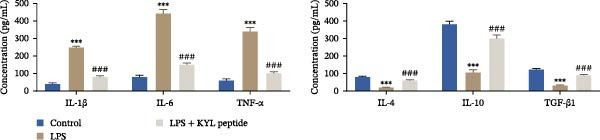
(A)

**Figure:**
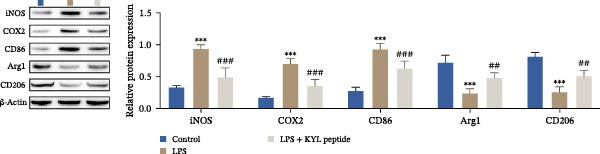
(B)

**Figure:**
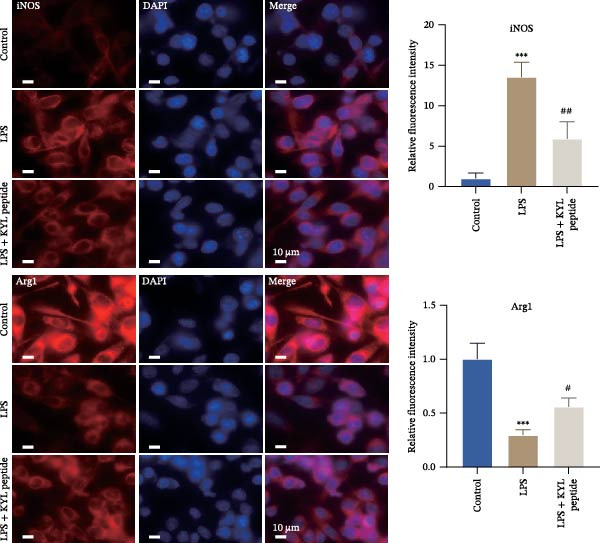
(C)

### 3.5. EPHA4 Promotes LPS‐Induced M1 Polarization of Microglia by Enhancing ERS

ERS is critical for microglial activation [9]. Therefore, we explored whether EPHA4 affects the M1 polarization of microglia by regulating ERS. Detection of ERS‐related proteins via Western blot revealed that the expression levels of p‐PERK/PERK, p‐IRE1α/IRE1α, ATF6, CHOP, and GRP78 were significantly upregulated in the brain tissues of TBI rats; after KYL peptide treatment, p‐PERK/PERK, p‐IRE1α/IRE1α, ATF6, CHOP, and GRP78 were partially downregulated. The effects of EPHA4 activator 123C4 on these proteins were opposite to that of the KYL peptide (Figure 5A). Ca^2+^ levels were detected by flow cytometry, which revealed that Ca^2+^ levels were significantly increased in the brain tissues of TBI rats. However, Ca^2+^ levels decreased after KYL peptide treatment and increased after 123C4 treatment (Figure 5B). Furthermore, LPS treatment also increased the expression of p‐PERK/PERK, p‐IRE1α/IRE1α, ATF6, CHOP, and GRP78 in GMI‐R1 cells. After 123C4 treatment, the expression of p‐PERK/PERK, p‐IRE1α/IRE1α, ATF6, CHOP, and GRP78 increased further, but these effects were weakened after treatment with the ERS inhibitor 4‐PBA (Figure 5C). Ca^2+^ levels were detected by flow cytometry. LPS treatment upregulated the Ca^2+^ level in GMI‐R1 cells, the Ca^2+^ level further increased after 123C4 treatment, and the Ca^2+^ level decreased after further 4‐PBA treatment (Figure 5D). ELISA results revealed that compared with the LPS group, 123C4 treatment further increased IL‐1β, IL‐6, and TNF‐α levels in GMI‐R1 cells and decreased IL‐4, IL‐10, and TGF‐β1 levels; these effects were weakened after further 4‐PBA treatment (Figure 6A). In addition, after 123C4 treatment, the expression levels of iNOS, COX2, and CD86 were further upregulated, and the expression levels of Arg1 and CD206 were further downregulated in GMI‐R1 cells. These results were weakened after further 4‐PBA treatment (Figure 6B,C). These results indicate that EPHA4 may promote the M1 polarization of microglia after TBI by activating ERS.

Figure 5EPHA4 promotes M1 polarization of microglia through the enhancement of ER stress. (A) Western blot detection of the ERS‐related proteins PERK, IRE1α, ATF6, CHOP, and GRP78 expressions in rat brain tissue; *n* = 5 rats/group. (B) Flow cytometry detection of Ca^2+^ levels in rat brain tissue; *n* = 5 rats/group. (C) Western blot analysis of the expression of the ERS‐related proteins p‐PERK/PERK, p‐IRE1α/IRE1α, ATF6, CHOP, and GRP78 in GMI‐R1 cells. (D) Flow cytometry detection of Ca^2+^ levels in GMI‐R1 cells. Compared with the sham or control group,  ^∗∗∗^ *p* < 0.001; compared with the TBI or LPS group, ^#^ *p* < 0.05, ^##^ *p* < 0.01, and ^###^ *p* < 0.001; compared with the LPS + 123C4 group, ^^^^^ *p* < 0.001.

**Figure:**
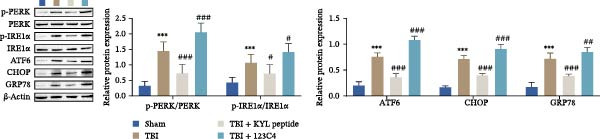
(A)

**Figure:**
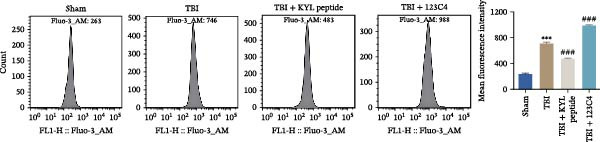
(B)

**Figure:**
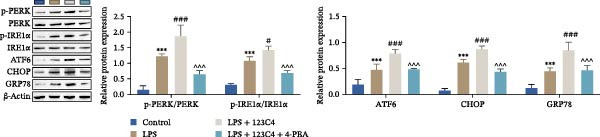
(C)

**Figure:**
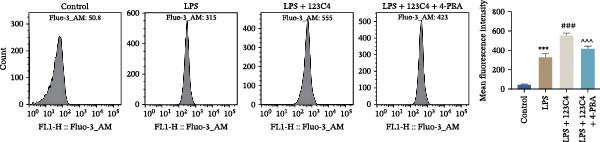
(D)

Figure 6EPHA4 promotes LPS‐induced M1 polarization of microglia through the enhancement of ER stress. (A) ELISA detection of IL‐1β, IL‐6, TNF‐α, IL‐4, IL‐10, and TGF‐β1 levels in GMI‐R1 cells. (B) Western blot detection of polarization markers in GMI‐R1 cells. (C) Immunofluorescence detection of iNOS and Arg1 expression in GMI‐R1 cells; scale bar: 10 μm. Compared with the control group,  ^∗^ *p* < 0.05,  ^∗∗^ *p* < 0.01,  ^∗∗∗^ *p* < 0.001; compared with the LPS group, ^###^ *p* < 0.001; compared with the LPS + 123C4 group, ^^^^ *p* < 0.01, ^^^^^ *p* < 0.001.

**Figure:**
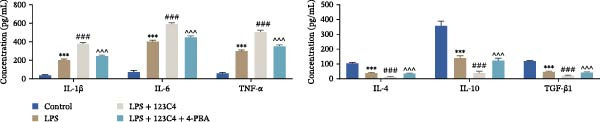
(A)

**Figure:**
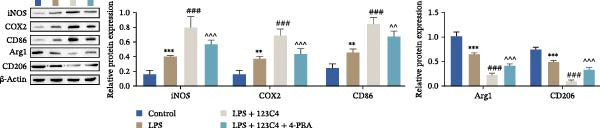
(B)

**Figure:**
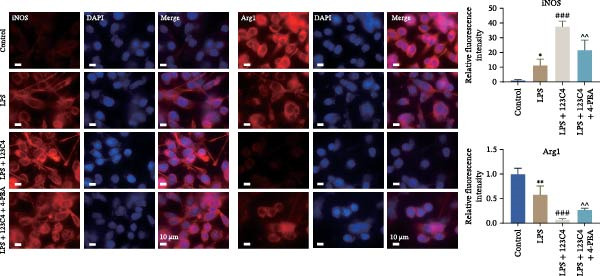
(C)

### 3.6. EPHA4 Activates MAPK Signaling in TBI Rats and LPS‐Induced Microglia

In addition, KEGG analysis based on the transcriptome sequencing results revealed that the MAPK pathway was significantly enriched in TBI (Figure 7A). Notably, previous studies have reported that the activation of the MAPK signaling pathway is regulated by EPHA4 [27]. Therefore, we explored the effect of EPHA4 on the activation of the MAPK signaling pathway in microglia. The expression of MAPK signaling pathway components was detected by Western blotting, which revealed that TBI or LPS promoted the expression of p‐ERK1/2/ERK1/2, p‐p38 MAPK/p38 MAPK, and p‐JNK/JNK in rat brain tissue or GMI‐R1 cells. Further KYL peptide intervention reduced the expression of these proteins (Figure 7B,C). These results indicate that EPHA4 can promote the activation of MAPK signaling in microglia.

Figure 7Activation of the MAPK signaling pathway by EPHA4 in TBI rats and LPS‐induced microglia. (A) KEGG enrichment analysis. (B) Western blot detection of the expression of p‐ERK1/2/ERK1/2, p‐p38 MAPK/p38 MAPK, and p‐JNK/JNK in the rat brain tissue; *n* = 5 rats/group. (C) Western blot detection of MAPK pathway‐related proteins in GMI‐R1 cells. Compared with the sham or control group,  ^∗∗∗^ *p* < 0.001; compared with the TBI or LPS group, ^##^ *p* < 0.01, ^###^ *p* < 0.001.

**Figure:**
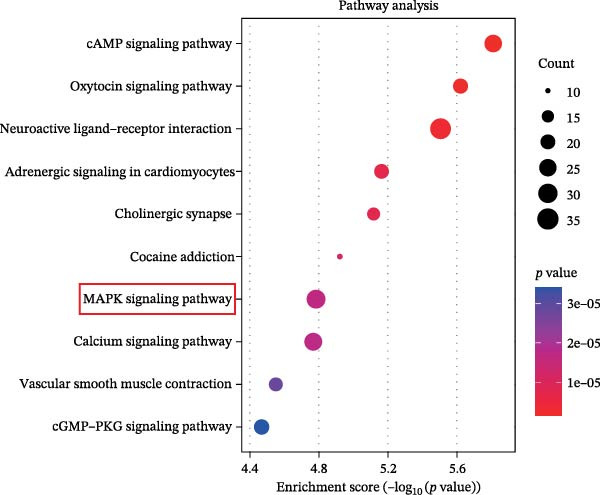
(A)

**Figure:**
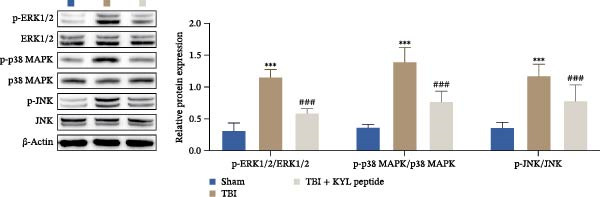
(B)

**Figure:**
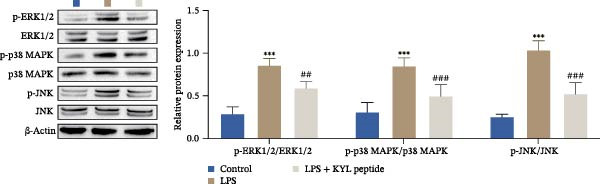
(C)

### 3.7. Activation of the MAPK Signaling Pathway Attenuates the Effects of EPHA4 Inhibition on ERS and M1 Polarization in Microglia

Previous studies have shown that MAPK signaling is also a key inducer of the occurrence of ERS after TBI [30]. Therefore, we explored whether the EPHA4/MAPK signaling pathway regulates ERS and affects M1 polarization in GMI‐R1 cells. We found that the effect of the KYL peptide was partially attenuated after C16‐PAF supplementation in GMI‐R1 cells, p‐ERK1/2/ERK1/2, p‐p38 MAPK/p38 MAPK, and p‐JNK/JNK (Figure 8A), and the p‐PERK/PERK, p‐IRE1α/IRE1α, ATF6, CHOP, GRP78 (Figure 8B), and Ca^2+^ levels (Figure 8C) were increased. ELISA results revealed that C16‐PAF treatment also attenuated the effect of the KYL peptide, increased the levels of IL‐1β, IL‐6, and TNF‐α, and decreased the levels of IL‐4, IL‐10, and TGF‐β1 in GMI‐R1 cells (Figure 9A). Furthermore, C16‐PAF treatment promoted the expression of iNOS, COX2, and CD86 and inhibited the expression of Arg1 and CD206 in GMI‐R1 cells (Figure 9B,C). These results indicate that EPHA4 may activate ERS by promoting the activation of the MAPK signaling pathway and promote M1 polarization of GMI‐R1 microglia.

Figure 8EPHA4 mediates ERS to promote M1 microglial polarization by activating MAPK signaling. (A) Western blot detection of the expression of p‐ERK1/2/ERK1/2, p‐p38 MAPK/p38 MAPK, and p‐JNK/JNK in GMI‐R1 cells. (B) Western blot detection of the ERS‐related proteins PERK, IRE1α, ATF6, CHOP, and GRP78 expression in GMI‐R1 cells. (C) Ca^2+^ level in GMI‐R1 cells detected by flow cytometry. Compared with the control group,  ^∗∗∗^ *p* < 0.001; compared with the LPS group, ^###^ *p* < 0.001; compared with the LPS + KYL peptide group, ^^^ *p* < 0.05, ^^^^ *p* < 0.01, ^^^^^ *p* < 0.001.

**Figure:**
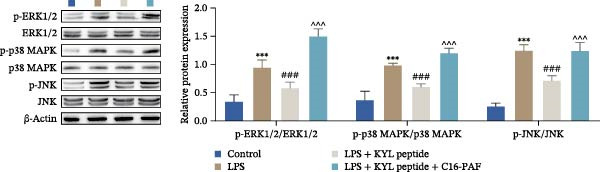
(A)

**Figure:**
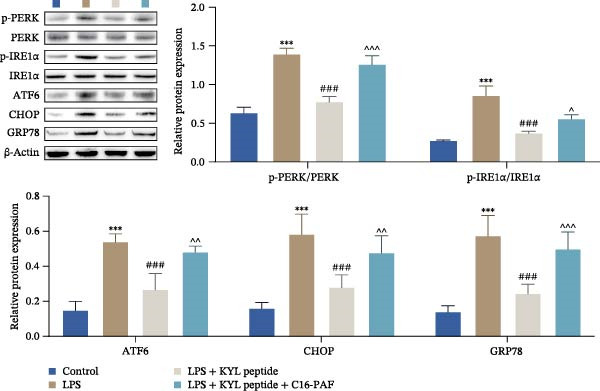
(B)

**Figure:**
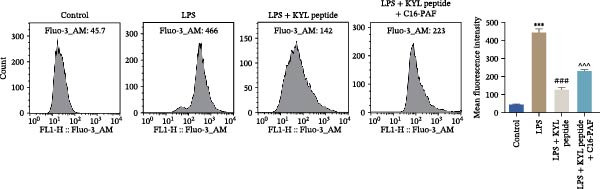
(C)

Figure 9EPHA4 mediates ERS to promote M1 polarization through activation of the MAPK signaling pathway. (A) ELISA detection of IL‐1β, IL‐6, TNF‐α, IL‐4, IL‐10, and TGF‐β1 levels in GMI‐R1 cells. (B) Western blot detection of polarization markers in GMI‐R1 cells. (C) Immunofluorescence detection of the expression of iNOS and Arg1 in GMI‐R1 cells; scale bar: 10 μm. Compared with the control group,  ^∗∗∗^ *p* < 0.001; compared with the LPS group, ^###^ *p* < 0.001; compared with the LPS + KYL peptide group, ^^^ *p* < 0.05, ^^^^ *p* < 0.01, ^^^^^ *p* < 0.001.

**Figure:**
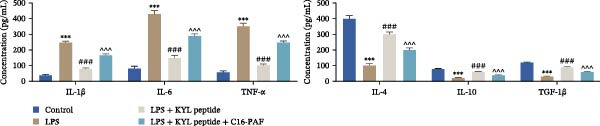
(A)

**Figure:**
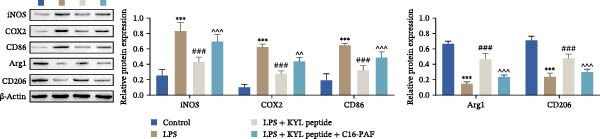
(B)

**Figure:**
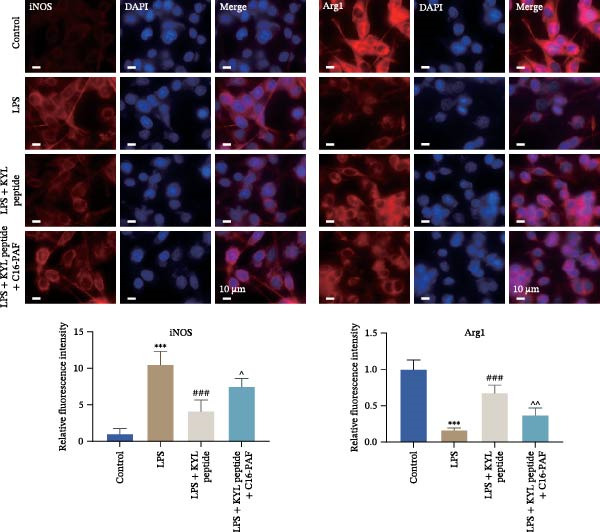
(C)

## 4. Discussion

TBI remains a leading cause of disability and death worldwide and is an important factor in long‐term cognitive impairment [31]. Neuroinflammation mediated by microglial is an important pathological event in TBI, and inhibition of microglial M1 polarization is considered to be an effective means to improve posttraumatic tissue damage and neural dysfunction [32]. Importantly, EPHA4 was upregulated in the brain tissue of rats with TBI, and inhibition of EPHA4 effectively attenuated M1 polarization in microglia and disease progression after TBI. Mechanistically, we found that EPHA4 may activate ERS in microglia by activating MAPK signaling, induce M1 polarization, and promote the progression of TBI. Our research has revealed the functions and regulatory mechanisms of EPHA4 in microglia after TBI, providing new potential targets and a theoretical basis for improving TBI.

To explore the key targets associated with the development of TBI, we used the brain tissue of TBI rats for transcriptome sequencing analysis and found that the expression of EPHA4 was upregulated after TBI. EPHA4 is a member of the erythropoietin‐producing human hepatocyte (Eph) receptor family, which is critical for embryonic brain development and axonal outgrowth during the early postnatal period [33]. More and more evidence indicates that EPHA4 is related to the brain injury response. For example, Frugier et al. [34] revealed that the upregulation of EPHA4 expression was associated with reactive astrocyte activation in human brain tissue after TBI. Greer et al. [18] showed that high levels of EPHA4 were also important causes of learning and memory impairment after TBI. Cash et al. [20] revealed that EPHA4 in TBI can induce BBB destruction, neural tissue damage, motor deficit, and cerebral blood flow injury through the negative regulation of endothelial cell function. Consistent with the results of previous studies, EPHA4 mRNA and protein expression levels were increased in the rat brain tissue after TBI. In TBI rats, after treatment with the EPHA4 inhibitor KYL peptide, the water content of the brain tissue decreased, and the integrity of the BBB and cerebral cortex damage were improved, indicating that inhibition of EPHA4 could delay the progression of TBI.

Furthermore, we found that on the third day after TBI in rats, EPHA4 was expressed in microglia (Iba‐1+) in the injured area and the surrounding regions. Combined with the time pattern of microglial polarization [28], these findings reveal a close relationship between EPHA4 and the activation of proinflammatory microglia. As important components of monocytes, microglia play key roles in the regulation of the immune microenvironment after TBI. Under normal conditions, microglia exhibit a relatively static surveillance phenotype and are continuously monitored in the brain parenchyma [35]. However, after TBI, the resting state of microglia is disrupted, and they differentiate into proinflammatory M1 type to induce a neuroinflammatory response, thus leading to neurodegeneration and neurological dysfunction [32]. In addition, Jiang et al. [36] showed that M1‐type microglia‐mediated neuroinflammation can also aggravate the progression of TBI by destroying the integrity of the BBB. Notably, Kowalski et al. [19] have confirmed that EPHA4 is also a novel regulator of monocyte/macrophage inflammation after LPS stimulation and TBI. Similar to the results of previous studies, we found that the levels of the proinflammatory cytokines IL‐1β, IL‐6, and TNF‐α were upregulated in the brain tissue of TBI rats and LPS‐induced GMI‐RI cells and that the levels of the anti‐inflammatory cytokines IL‐4, IL‐10, and TGF‐β1 were downregulated, and the level of M1‐type microglial polarization was upregulated. The effects of TBI or LPS could be partially reversed by the KYL peptide. Our study revealed a key role for EPHA4 in promoting the progression of TBI through the promotion of microglial M1 polarization.

ER is an important organelle for protein synthesis, folding, modification, and Ca^2+^ homeostasis within the cells. However, under the influence of pathological injury, such as TBI, the normal function of the ER is impaired, which leads to the accumulation of misfolded and unfolded proteins, resulting in ERS [37]. Persistent ERS is also an important factor contributing to TBI‐associated apoptosis and neural dysfunction [12, 38]. Wu et al. [39] revealed that ERS inhibition effectively improved TBI‐induced BBB injury. In addition, ERS is critical for microglial activation and the subsequent neuroinflammatory response [9]. Therefore, we explored whether EPHA4 affects the M1 polarization of microglia in TBI by regulating ERS. We found that the expression of the ERS‐related proteins p‐PERK/PERK, p‐IRE1α/IRE1α, ATF6, CHOP, and GRP78 was upregulated and that the Ca^2+^ level was increased in TBI rats brain tissues and LPS‐induced GMI‐R1 cells, and 123C4 treatment further enhances ERS and increases the level of Ca^2+^, whereas 4‐PBA treatment reduced the levels of ERS and Ca^2+^. In addition, LPS treatment upregulated the expression of iNOS, COX2, and CD86 and the levels of IL‐1β, IL‐6, and TNF‐α in GMI‐R1 cells and downregulated the expression of Arg1 and CD206 and the levels of IL‐4, IL‐10, and TGF‐β1. 123C4 treatment further enhanced the effect of LPS, while the additional 4‐PBA weakened the effect of 123C4. Our study revealed that EPHA4 could promote the M1 polarization of microglia after TBI by activating ERS.

To further clarify the signaling pathways associated with EPHA4, we performed KEGG pathway analysis on the basis of the transcriptome sequencing results and found that the MAPK pathway was significantly enriched. MAPK signaling involves three pathways, namely, the JNK, p38, and ERK pathways, in addition to mediating cell proliferation, differentiation, apoptosis, and inflammation [22], and is involved in pathological changes after TBI [7]. For example, Zhang et al. [40] revealed that the phosphorylation of p38 MAPK and ERK1/2 is associated with the development of pathological events such as neurological dysfunction, cerebral blood flow, and reduced neural blood flow in TBI rats. Celorrio et al. [41] have shown that the MAPK signaling pathway is also a key regulatory pathway for fear memory and hypoxemia after TBI. Lan et al. [42] confirmed that the phosphorylation of p38, ERK, and JNK induces cognitive dysfunction in TBI rats through the activation of inflammation. Additionally, Wang et al. [27] have shown that EPHA4 can affect the fate of NSCs by regulating the activation of the MAPK signaling pathway. In this study, LPS treatment promoted the expression of p‐ERK1/2/ERK1/2, p‐p38 MAPK/p38 MAPK, and p‐JNK/JNK in GMI‐R1 cells, whereas KYL peptide intervention decreased these proteins expression. Notably, MAPK signaling pathway is also involved in TBI‐induced ERS [30]. Therefore, we further explored whether EPHA4 affects ERS after TBI by regulating MAPK signaling and the effect of this regulation on the M1 polarization of microglia. In this study, the positive effect of the KYL peptide was partially attenuated by the MAPK activator C16‐PAF, resulting in increased p‐ERK1/2/ERK1/2, p‐p38 MAPK/p38 MAPK, p‐JNK/JNK expression, ERS level, Ca^2+^ level, inflammatory level, and M1 polarization level of GMI‐R1 cells. Our study revealed that EPHA4 may activate ERS by promoting MAPK signaling, thereby promoting M1 polarization of microglia after TBI.

Although encouraging results have been achieved, this study has some limitations. First, based on previous studies, we used an osmotic pump to administer the KYL peptide into the rats. The results of the study were in line with our expectations. However, there was no evidence to suggest that this peptide could effectively reach the brain parenchyma at a functional concentration. Furthermore, while the KYL peptide has been used to regulate EPHA4 function in various biological systems [43], the existence of off‐target effects of the KYL peptide remains unknown. This poses a challenge for future research. It will be necessary to explore in the future whether the drug delivery method using osmotic pumps can completely deliver the KYL peptide to the brain and whether the KYL peptide has specificity for EPHA4. Second, the dosage of the agents (such as 123C4 and C16‐PAF) used in this study was based on previous research. Although the experimental results were in line with expectations, the exact reference for each compound was not always clearly linked. Therefore, future research still needs to further evaluate the optimal dosage of these agents in TBI studies.

In conclusion, our study highlights the potential of EPHA4 as an intervention target to improve the M1 polarization of microglia after TBI. Importantly, we elucidated the key mechanism by which EPHA4 may activate ERS by activating MAPK signaling, induce M1 polarization of microglia, and ultimately promote TBI progression. These findings greatly increase our understanding of the function of EPHA4 in M1‐type microglia‐mediated inflammation and TBI.

## Author Contributions

Conceptualization: Yang Tan and Chuanyun Qian. Formal analysis: Jing Xia and Haiying Wu. Methodology: Yang Tan. Data acquisition: Yang Tan and Mingwei Liu. Project administration: Chuanyun Qian. Resources: Haiying Wu. Software: Sangyang Deng. Supervision: Yang Tan. Validation: Jing Xia and Sangyang Deng. Visualization: Mingwei Liu. Writing – original draft: Yang Tan. Writing – review and editing: Chuanyun Qian. Funding acquisition: Chuanyun Qian.

## Funding

The study was supported by the National Natural Science Foundation of China (Grant 82260384), the project team of the Medical Center of Yunnan Province in 2025 (Grant 2024YNLCYXZX0079), research on the Mechanism and Prevention of High‐Risk Toxic Biological Poisoning in Yunnan (Grant 2024YNLCYXZX0083), and Grants from the Yunnan Province Science and Technology commission (Grant 202305AF150186).

## Disclosure

All authors contributed substantially to this manuscript, and all authors read and approved the final manuscript.

## Ethics Statement

All animal experiments were approved by the Animal Experiment Ethics Committee of Kunming Medical University (kmmu20211255), and all animal experimental protocols were adhered to the ARRIVE guidelines 2.0.

## Conflicts of Interest

The authors declare no conflicts of interest.

## Data Availability

The data are available from the corresponding author upon reasonable request.
